# Prediction-Based Human-Robot Collaboration in Assembly Tasks Using a Learning from Demonstration Model

**DOI:** 10.3390/s22114279

**Published:** 2022-06-03

**Authors:** Zhujun Zhang, Gaoliang Peng, Weitian Wang, Yi Chen, Yunyi Jia, Shaohui Liu

**Affiliations:** 1 State Key Laboratory of Robotics and System, Harbin Institute of Technology, Harbin 150001, China; 14b908005@hit.edu.cn; 2 Department of Automotive Engineering, Clemson University, Greenville, SC 29607, USA; wangw@montclair.edu (W.W.); yc4@g.clemson.edu (Y.C.); yunyij@clemson.edu (Y.J.); 3 School of Computer Science and Technology, Harbin Institute of Technology, Harbin 150001, China; shliu@hit.edu.cn

**Keywords:** human-robot collaboration, action prediction, assembly, spatiotemporal, deep learning, human demonstration, robot learning

## Abstract

Most robots are programmed to carry out specific tasks routinely with minor variations. However, more and more applications from SMEs require robots work alongside their counterpart human workers. To smooth the collaboration task flow and improve the collaboration efficiency, a better way is to formulate the robot to surmise what kind of assistance a human coworker needs and naturally take the right action at the right time. This paper proposes a prediction-based human-robot collaboration model for assembly scenarios. An embedded learning from demonstration technique enables the robot to understand various task descriptions and customized working preferences. A state-enhanced convolutional long short-term memory (ConvLSTM)-based framework is formulated for extracting the high-level spatiotemporal features from the shared workspace and predicting the future actions to facilitate the fluent task transition. This model allows the robot to adapt itself to predicted human actions and enables proactive assistance during collaboration. We applied our model to the seats assembly experiment for a scale model vehicle and it can obtain a human worker’s intentions, predict a coworker’s future actions, and provide assembly parts correspondingly. It has been verified that the proposed framework yields higher smoothness and shorter idle times, and meets more working styles, compared to the state-of-the-art methods without prediction awareness.

## 1. Introduction

Innovations in design and control technology are allowing various robots to complete complicated tasks in wide segments of our life. Increasingly sophisticated robots have been crafted to populate different areas. With the booming of computer science and artificial intelligence, social robots [1] utilize extensive sensors and massive data to gradually develop human-like communication styles and adjacent safety capabilities. There is evidence that the same trends have begun to permeate industry [2] for specific assignments where requirements were previously off-limits. Henceforward, robots are expected to interact with humans in more challenging aspects, such as the assembly process.

The traditional assembly process [3] can be exhausting for humans, due to the bulky payload, repetitious procedures, and possible hazardous conditions. Nowadays, many six-axis robots are used in automation manufacturing [4] to achieve higher capacity and better quality. Compared with humans, robots can handle the payload effortlessly and carry out the repeated work tirelessly. These industrial robots relieve humans from monotonously repetitive work. However, small and medium-sized enterprises (SMEs) have surpassed large corporations and become the backbone of most industrialized countries [5]. They typically provide new, pioneering products with flexible supply chains for serving different industries and responding to rapid changes in market demands. As the requirements of the product keep changing, the supplier environment building and integration must evolve [6] in a similar manner. In fact, SMEs are evolving far more rapidly than ever before, owing to the advantages of automation and robotics.

As production trends are rapidly changing, the main trigger points in intelligent manufacturing [7] are starting to arise, namely non-standard and flexibility. In reality, it is often difficult to anticipate every possibility and develop fully scheduled responses accordingly for every situation with the limited resources of robots. Taking the economy under consideration, not all tasks can be automated to solely rely on robots, and some tasks are too expensive to achieve automation without help from humans. Since the intelligence of robots [8] will not be sufficient in the near future to carry out various expectations independently, humans have to engage in these procedures with their flexibility and cognition strength. 

To deal with these issues, the deployed robots must be able to change assignments easily and be able to work alongside [9] humans. Therefore, the use of human-robot collaboration (HRC) [10] fits the bill properly in current and future manufacturing scenarios. Particularly, for assembly manufacturing applications, collaborative robots can be deployed to assist diverse assignments using cooperative motion control methods. As a result, humans are less involved in these projects, but their involvement becomes more crucial for the performance of the whole system. 

Recently, many developers have improved HRC technologies [11] to meet the increasing requirements for assembly applications. During human collaborative assembly, we can infer our working peer’s social cues and engage in these complex activities cognitively. Similar capabilities should be adapted to the robotic assembly coworkers, which can attain essential information from observation [12] and play the corresponding role in line with cooperative humans. By involving humans in the robot workspace, a lot of difficulties are introduced for this field research, i.e., learning from demonstration [13,14], human intention recognition [15], human-aware motion planning [16], visual servoing [17], etc. With the rise of collaborative robots, a lot of research is being conducted on safety as well. Scalera et al. [18] presented a safety control scheme based on the standards of protective separation distance and bounding volume for collaborative application. The dynamic safety zones are applied to design the collision avoidance strategy. Himmelsbach et al. [19] proposed to extend human-machine differentiation to the safety system in the human-robot collaboration. Their method treats the differentiation of human and non-human objects carefully to maintain efficiency while decreasing the protective separation distance. The collaborative robots need to understand the actions occurring and infer the coworker’s intentions swiftly, to deliver the correct parts to the human at the right time. Since the planning and execution are time-consuming for robots in the collaboration space, it is critical for the robot to understand the evolving tasks within the unstructured workspace [20], predict the counterpart’s intention in an advanced window, and adjust the related actions automatically. To design a corresponding system to achieve this function, it is critical to establish a learning framework for human action early prediction and that is the cornerstone of this work.

Early research on the prediction of human motions was generally based on heavy handcrafted models to heuristically arrange the predefined processes. Mainprice et al. [21] proposed a gesture recognition-based framework to generate a workspace occupancy prediction, then the planner plans the optimal trajectory considering the prediction. Matteo Ragaglia et al. [22] illustrated a trajectory scaling algorithm for collaboration subjected to human occupancy prediction. They converted the problem into a series of convex polytopes by evaluating the reachable sets for each degree of freedom. However, the whole assembly procedures are generally quite complicated to form for the users. The model-based approaches [23] require expert knowledge to design and debug for the collaboration. Furthermore, the human part introduces tremendous uncertainties [24] into the collaborative assembly process, which considerably increases the effort to adapt and maintain the model in diverse situations. 

There were various studies adapted from the statistics-related method in the human action prediction area. Hawkins et al. [25] designed a collaborative activity representation in linear chains with conditional dependency. Then, the sub-tasks were predicted in a probabilistic manner. Hongyi Liu et al. [26] presented a hidden Markov model to predict human motions in a human-robot collaborative system. They formed an assembly task as a sequence of human motions and then recognized them to obtain the motion transition probabilities. Zanchettin et al. [27] schemed a method to predict human activity patterns in a small part collaborative assembly task. The higher-order Markov chains algorithm was deployed to achieve the prediction process. Lin et al. [28] developed a hidden semi-Markov model to predict the human assembly rate for human-robot collaboration. Their model links the human hidden risk perception with the visible assembly rate and adjusts the robot’s response according to the predicted human behavior. A competitive fluent assembly process with a shorter waiting time for the operator is achieved in their experiment scenarios. However, in the assembly process, the future action may not only depend on the current action. The nature of the assembly process restrains the limitations of prediction. For example, the future action may depend on several early operations, or even on the entire assembly sequence. The model needs to be able to make the appropriate prediction based on long-term historical information in the collaborative assembly applications. 

Some works rely on the recognition of human gestures to classify and make reasonable predictions about the actions performed by a human. Luo et al. [29] used a two-layer framework of Gaussian mixture models to recognize and predict human reaching motions. The model observed the trajectory, determined which class it belongs to, and predicted the remainder of the trajectory. Huang et al. [30] devised an anticipatory control method to assist human partners based on observed gaze patterns. They classified the gaze patterns using a support vector machine and predicted the potential requested item. Buerkle et al. [31] presented an upper-limb movement intention prediction model with a mobile electroencephalogram. They classified the signals with a recurrent neural network model in a semi-online system. Liu et al. [32] combined temporal processing with the convolutional neural network to predict human motion in the disassembly tasks. They attained motion recognition through deep learning and performed the prediction based on the recognition results. However, generally in the assembly procedures, the recognition-based methods usually require a lot of effort to construct the supportive setups and the complex predefinitions of the tasks. These challenges also increase the deployment expense and restrict customized manufacturing.

Some studies try to estimate human intention through various sensors other than vision sensors. Muthugala et al. [33] proposed a system to identify the user intention for the interpretation of ambiguous instructions related to distances in robot navigation scenarios. The actual intention is identified by analyzing the gesture information and voice instructions through a rule-based fuzzy method. Luo et al. [34] presented a framework to infer human intention and predict human hand motion. The training data were collected by a Kinect device. Probabilistic Dynamic Movement Primitive is applied for the hand trajectory prediction in a manipulation task. Wang et al. [35] developed a teaching-learning-prediction model for the robot to predict human hand-over intentions using wearable sensors. The extreme learning machine algorithm is applied to infer human intention from partial demonstrations. Casalino et al. [36] proposed a framework to infer human intentions through a Bayesian recursive classifier. The position of the head and hand is exploited to predict human behavior. They also achieve human awareness in the collaborative task by equipping the wearable device with haptic feedback. 

Despite the different methods in various intention identification applications, the data source is also miscellaneous in human-robot collaboration. In general, wearable sensors provide more condensed information than vision sensors. Remote status monitoring provided by wearable technology has become an important tool in the industrial collaboration scenario. Furthermore, wearable devices can provide real-time feedback regarding collaboration conditions. 

However, wearable devices may introduce inconvenience during the assembly procedure, while vision-based methods are non-intrusive. The vision-based methods can offer natural, contact-free interactions. In addition, vision sensors have the advantage of deployment simplicity and low cost compared to wearable devices. Furthermore, vision-based methods can provide sufficient high-dimension data for the system and sustain flexibility with extensibility for future applications. Since the collaborative actions may vary spatiotemporally with assorted implications, the vision-based methods can enable human-robot collaboration that is difficult or impossible to fulfill with other modalities. In the meantime, the vision-based method can be enhanced through wearable feedback in multimodal settings.

A comparison of the representative methods in human action prediction is presented in Table 1. It shows our method is suitable for the current human prediction difficulties. To address these challenges, we propose a human action prediction model based on learning from demonstration in this paper. The proposed method allows robots to learn undefined tasks within the shared workspace from human demonstrations, and to predict human actions in advance. Performing this allows trained robots to proactively assist humans early on, thus making the collaborative process in collaborative assembly smoother and more efficient. By exploiting the assistance of trained robots, the advantage of humans and robots can be combined in the collaborative assembly scenario. Human workers can get the right component at the right time according to their unique assembly habits. Additionally, human workers no longer need to search for components on a large number of shelves but can focus on the subtler assembly processes. Finally, prediction-based collaboration can bring improvement in terms of efficiency and quality, which is the goal of intelligent manufacturing. 

The main contributions of this work can be summarized as follows: (1) We propose prediction-based learning from a demonstration framework for robots to understand assignment descriptions and human customized working preferences toward improved human-robot collaboration. (2) We extend the ConvLSTM concept and develop a novel state-enhanced ConvLSTM model for robots to learn spatiotemporal dependency and predict long-term action dynamics efficiently in collaborative works. (3) We establish a tracking result guided CNN model to leverage the prediction knowledge to proactively assist humans according to their preferences. 

The remainder of this paper is organized as follows. After the discussion of the related work, we describe the proposed framework and necessary theory in Section 2. Section 3 contains the experiment settings, and Section 4 exhibits a review of important results from the experiments. The conclusion is presented in Section 5.

## 2. Materials and Methods

### 2.1. A Brief Overview of the Prediction-Based Framework

**Figure 1:**
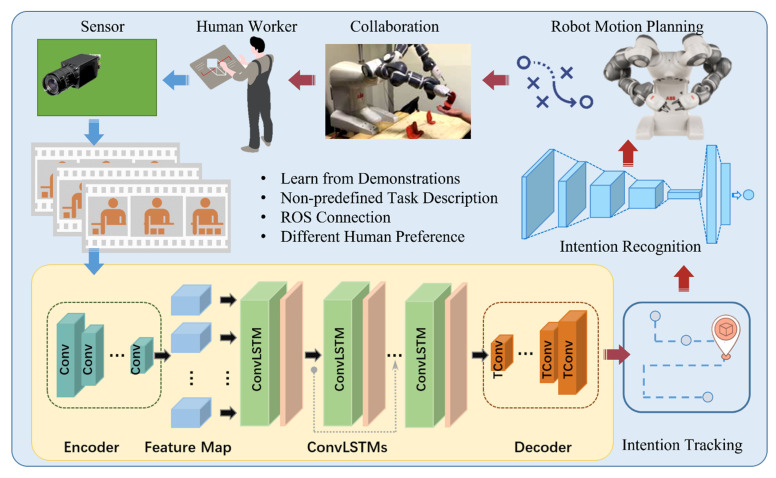
Prediction-based human-robot collaboration framework.

The purpose of this paper is to predict a human worker’s intentions early and fulfill the human-robot collaboration properly for the assembly scenarios. The gist of the proposed framework is shown in Figure 1. The data of assembly tasks and workspace evolvements are collected by the vision sensor. Then, the raw data are prepared and preprocessed for the subsequent pipeline. After the pretreatment, the gathered input assembly information is fed into the recurrent neural network module for intention prediction. This recurrent neural network was trained based on learning from human demonstrations. During the training process, the relevant assembly information passes down the action sequences to tune the parameters. The recurrent neural network [37] learns to keep the essential knowledge, forget the unimportant facts, and make helpful predictions. The output from the human collaboration intention prediction module goes through a tracking module based on an extended Kalman filter [38] to obtain the specific intended assembly part. The acquired intended part is recognized in the following deep learning module. Finally, the recognized intended assembly part provokes the robot’s motion planning system [39], and the robot executes the picking up and delivering action at the appropriate timing for future assembly collaboration.

The framework only shows the main data flow and information process pipeline, while omitting the ROS connection system [40] and robot planning part. The main sub-system’s details are introduced in the following sub-sections of this section, which include the learning and prediction model, intention parts tracking model, and intention recognition model. 

### 2.2. Learning from Demonstration and Human Intention Prediction

The proposed framework uses an end-to-end recurrent neural network to implement human demonstration learning and human intention prediction. Specifically, the ConvLSTM [41] was applied to handle the spatial and temporal information during the assembly process. 

The ConvLSTM layer can be explained by the equations below:

(1) $$\begin{array}{l} i_{t}=\sigma (W_{xi}*X_{t}+W_{hi}*H_{t-1}+W_{ci}\circ C_{t-1}+b_{i})\\ f_{t}=\sigma (W_{xf}*X_{t}+W_{hf}*H_{t-1}+W_{cf}\circ C_{t-1}+b_{f})\\ C_{t}=f_{t}\circ C_{t-1}+i_{t}\circ \tanh(W_{xc}*X_{t}+W_{hc}*H_{t-1}+b_{c})\\ o_{t}=\sigma (W_{xo}*X_{t}+W_{ho}*H_{t-1}+W_{co}\circ C_{t}+b_{o})\\ H_{t}=o_{t}\circ \tanh(C_{t}) \end{array}$$

where *X_t_* is the input sequence at time *t* and equals the output from the encoder. W_**_ and b_**_ serve as the weight matrices and bias terms. *σ* indicates a sigmoid function, and tanh denotes the hyperbolic tangent function. *i_t_*, *f_t_*, *o_t_* represent the input gate, forget gate, and output gate, respectively. C_*_ and H_*_ refer to the cell unit and hidden state. ‘*’ represents the convolution operator and *‘o’* represents the Hadamard product operator.

Compared to the fully connected LSTM [42], ConvLSTM introduces convolutional structures in both the input-to-state and state-to-state transitions. However, the association between these two parts in ConvLSTM is relatively insufficient in complex models. More important details disappear as the data pass through more iterations. There are more couplings between the *H* state and input *X* needed to be designed to keep more useful information in the challenging models. 

We add some extra linkages in the model to extract the essential relations for the long-time forecasting performance. The linkages make the coupling of *H*_*t*−1_ and *X_t_* richer to keep persistent knowledge between network layers and reduce information loss as the time sequence expands. Similar to the filter process, we take advantage of the convolutional approach to build a feature map and constrain the network hidden states with this map. In this way, more crucial spatiotemporal information will be sustained in the model during the sequence processing.

Specifically, the linkage is described in the following equations:

(2) $$\begin{array}{l} i_{t}=\sigma (W_{xi}*X_{t}+W_{hi}*H_{t-1}^{\prime }+W_{ci}\circ C_{t-1}+b_{i})\\ f_{t}=\sigma (W_{xf}*X_{t}+W_{hf}*H_{t-1}^{\prime }+W_{cf}\circ C_{t-1}+b_{f})\\ C_{t}=f_{t}\circ C_{t-1}+i_{t}\circ \tanh(W_{xc}*X_{t}+W_{hc}*H_{t-1}^{\prime }+b_{c})\\ o_{t}=\sigma (W_{xo}*X_{t}+W_{ho}*H_{t-1}^{\prime }+W_{co}\circ C_{t}+b_{o})\\ H_{t-1}^{\prime }=\sigma (W_{xh}*X_{t}+b_{xh})\circ H_{t-1}\\ H_{t}=o_{t}\circ \tanh(C_{t}) \end{array}$$

where *H*_*t*−1_ is the hidden state in the previous equation, and all the following *H*_*t*−1_ parts are replaced with the state-enhanced $H_{t-1}^{\prime }$. *W_xh_* and *b_xh_* serve as the corresponding weight and bias terms. ‘*o*’ represents the Hadamard product operator as before. Other terms remain the same as in Equation (1).

As the data flow through the ConvLSTM network, more and more details fade away due to the information decay. We add the additional operations to learn more global and local patterns in the input data which have temporal properties. Then, the input-related feature map treated by the Hadamard product with the hidden state also helps to improve prediction performance since the operations can be considered as the regularization in the sequence processing. This can extract spatiotemporal details more effectively using combinations of input and state data flow. The state enhancement procedure can be deployed multiple times like the convolutional layers. 

In order to improve the efficiency of the proposed model, a convolutional encoder [43] is used to abstract the essential features of the input sequence. Then, the obtained features are fed into the ConvLSTMs with state enhancement to calculate the cell states and keep the critical spatial and temporal knowledge. Next, a convolutional decoder is attached to generate predictions given the output from the previous layers. 

The complete learning and prediction framework can be explained by Equation (3):

(3) $$\begin{array}{l} X_{1,\ldots ,t}=\mathrm{CNNEncoder}(I_{1},I_{2},\ldots ,I_{t})\\ \left(H^{n},C^{n})={\mathrm{ConvLSTM}}_{k}(X_{1,\ldots ,t},C^{n-1}\right)\\ \left(Y_{t+1},Y_{t+2},\ldots ,Y_{t+T})=\mathrm{CNNDecoder}(H^{n}\right) \end{array}$$

where *X*_1,…,*t*_ represents the feature maps extracted from the convolutional encoder. *I*_1_, *I*_2_, …, *I_t_* represents the input sequence. *H^n^* and *C^n^* represent the hidden and the cell states of ConvLSTMs with state enhancement. *Y*_*t*+1_, *Y*_*t*+2_, …, *Y_t+T_* stands for the predicted frames from the convolutional decoder. This model can be tuned by applying different layers of encoder/decoder and ConvLSTMs in accordance with various situations. 

### 2.3. The Predicted Part Tracking System Design

An extended Kalman filter (EKF)-based tracking algorithm is proposed here to track the human intention part. The Kalman filter [44] has been used successfully in some industry systems such as robot navigation and object tracking. The Kalman filter applies a set of recursive equations to calculate the optimal system state efficiently by using a form of feedback control. The EKF is a nonlinear variation of the Kalman filter. However, the EKF is not an optimal estimator generally, instead it gives an approximation of the optimal state estimate by linearization.

The EKF equations are shown below:

(4) $$\begin{array}{l} {\hat{x}}_{k}^{-}=f({\hat{x}}_{k-1},u_{k-1},0)\\ P_{k}^{-}=A_{k}P_{k-1}A_{k}^{T}+W_{k}Q_{k-1}W_{k}^{T}\\ K_{k}=P_{k}^{-}H_{k}^{T}{\left(H_{k}P_{k}^{-}H_{k}^{T}+V_{k}R_{k}V_{k}^{T}\right)}^{-1}\times \\ {\hat{x}}_{k}={\hat{x}}_{k}^{-}+K_{k}(z_{k}-h({\hat{x}}_{k}^{-},0))\\ P_{k}=(I-K_{k}H_{k})P_{k}^{-} \end{array}$$

where the first two equations show the state and covariance estimates from the previous time step *k* − 1 to the current time step *k*. ${\hat{x}}_{k-1}$ is an a posteriori estimate of the state, *u*_*k*−1_ is the driving function, and *f* is the non-linear function. *Q_k_* is the process noise covariance. *A_k_* and *W_k_* are the process Jacobian matrix at step *k*. The following three equations show the correction process for state and covariance estimates with the measurement update. *P_k_* is the estimation error covariance. *H_k_* and *V_k_* are the measurement Jacobians at step *k*, and *R_k_* is the measurement noise covariance at step *k*. *h* stands for the non-linear measurement-related function. *z_k_* represents the measurement, and *K_k_* represents the Kalman gain.

Besides the non-linear estimation problem, there are several challenges for the intended object tracking in the collaboration scenario. For instance, the position of the intended object may be measured with error, the intended object may be occluded partly by a human hand, and a new intended object may appear in the tracking area. To address these challenges, the EKF was utilized in our tracking system. 

After initialization and parameter tuning, the system will make a state prediction based on previous parameters and the model. Then, the predicted position and the measured position will be combined to update the system. Meanwhile, the EKF will change the weight ratio depending on the uncertainty. This process repeats as the tracking evolves. The EKF-based tracking system outputs the tracked position of the former prediction robustly. Moreover, the intended parts can be cropped out according to the tracking position. 

### 2.4. The Assembly Intention Recognition System Design

Once these images of intended parts are available, the next step is to recognize the specific part and prepare the robot to collaborate with the human in the assembly scenario. During the tracking process, the type and the shape of the intended part could be difficult to handle for the traditional methods [45]. A convolutional neural network (CNN) model [46] is designed to recognize the intended assembly part with high accuracy in our framework. 

The model accepts tracking input images to abstract the feature map with the convolutional operation. With the process of several convolution layers and pooling layers, features of different levels can be captured for further computation. These features are fed into the fully connected layers for classification. The probability of each class is calculated by the softmax function. Finally, the model uses the cross-entropy loss function to evaluate the consistency of probability and the objective function.

The process can be explained in the following abbreviated equations:

(5) $$\begin{array}{l} Y=f(W\times X+b)\\ q=\mathrm{softmax}(Y^{\prime })\\ Loss=-\sum p\log(q) \end{array}$$

where *X* stands for the input of each convolutional layer, *W* and *b* stand for the weight and bias terms to be trained for each layer. *Y′* represents the feature map output from the convolutional layers and pooling layers. *q* represents the probability of the output class. *p* shows the objective one-hot vector for the cross-entropy loss function. As for the activation function, we use a rectified linear unit (ReLU) in the model. To improve the efficiency and performance of the model, the batch normalization approach [47] is added to our model as well.

Generally, moving parts recognition can be particularly tricky while facing complex assembly situations. The learning CNN model can ease this dilemma with its decent generalization performance. The final collaboration decision can be settled conveniently for different task arrangements and various assembly preferences.

## 3. Experiment Settings 

### 3.1. An Overview of Learning from Demonstration Approach

**Figure 2:**
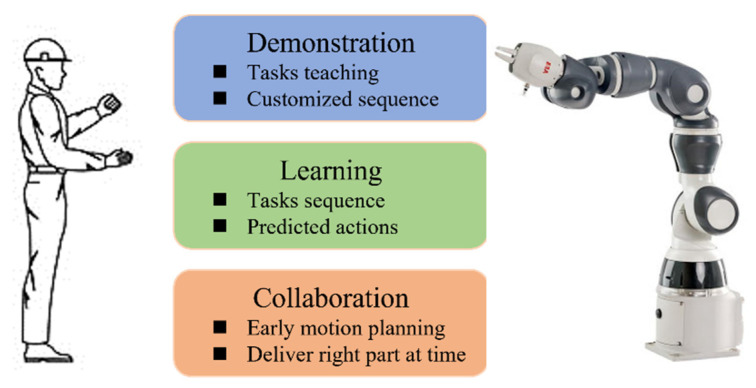
The scheme of the learning from demonstration model.

In the human-robot collaborative assembly process, robots are required to assist humans dynamically. Therefore, in the context of the assembly, robots controlled by rigid codes are undesirable due to the incapacity to adapt to changing situations and the need for expert knowledge. We proposed a learning from demonstration based model to handle this problem. The robot can be programmed by human workers without expert knowledge. The collaborative task arrangement can be transferred to the robot in a natural and expressive way. The diagram of this model is shown in Figure 2. 

In the demonstration phase, the human worker acts like an experienced teacher for the robot. During the human-robot interaction process, the complex tasks and customized assembly styles may be difficult to be defined as a known function, but they can be demonstrated directly. The robot collects the assembly information from the sensors while the human performs the assembly assignments. With the human demonstration, the robot will learn what is going on in the shared workspace. As in the learning process, the robot learns the task details implicitly and analyzes the optimal actions naturally without increasing the programming burden. This process will eliminate the need for expert knowledge-oriented and time-consuming methods. The robot accomplishes a complex mapping to the human demonstration considering the constraints from the shared workspace and the requirements from the assembly actions. After the robot obtains the fundamental experience, various applications can be realized in different scenarios. Here, we will focus on the assembly collaboration circumstance, i.e., how to make early plans to assist humans by delivering parts at the right time. In this project, a state-enhanced recurrent neural network based model is introduced to learn the task description and arrange proper executions to cooperate with the human worker while improving the fluency and safety of the human–robot collaboration.

### 3.2. The Assembly Signal Collection Process 

**Figure 3:**
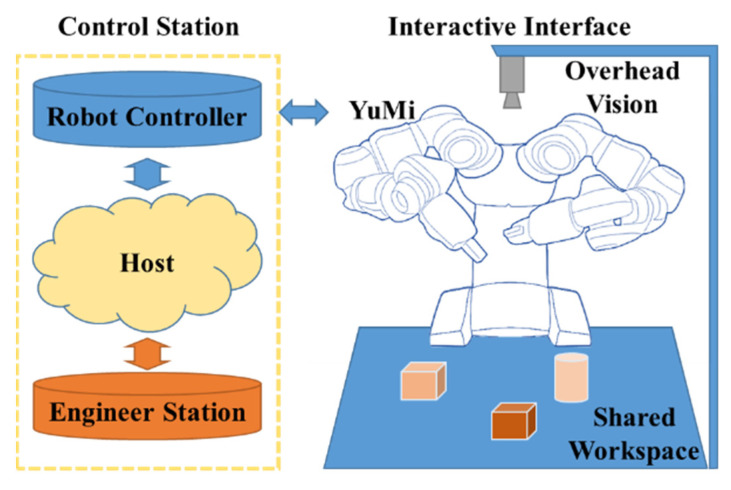
The collaborative research platform.

In this work, the signal was collected from the collaborative research platform, as shown in Figure 3, which contains a YuMi robot, a control station, and an expansible interactive interface. The payload of the YuMi robot is 0.5 kg per arm. This payload is ideal for small parts assembly system prototype design and verification. Due to the payload limit of the YuMi robot, a downscale vehicle seats assembly experiment environment was set up. 

Humans can gather assembly information through their eyes and efficiently figure out the proper manner to cooperate with other coworkers. Hence, the natural way to collect assembly information for the robot is through cameras. The main purpose of this study is to build a prediction-based human–robot collaboration model for assembly manufacturing. During the assembly process, humans will make corresponding actions according to the assembly goal in the shared workspace. The robot should focus on the ongoing actions within the workspace and the motion of human hands [48] is a significant part of all these complex actions. The future assembly intentions and action predictions can be obtained from the perceived history of human hand manipulation actions. With the matching parts in human hands, the assembly process can be achieved successfully. Therefore, the assembly signal collection of this study concentrates on the movement of the parts within the collaboration workspace. Although we only consider the perception and prediction of hand actions in this project, the framework can be easily applied to a wider range of motion predictions such as arms and torso in the workspace. The real-time image series data from the overhead vision sensor were sampled at a frame rate of 10 Hz and then fed into the preprocessing system. The data collection stage prepares the formatted input for the prediction system and other associated systems during the human-robot collaborative assembly.

### 3.3. The Analysis Procedure of Assembly Tasks 

In this project, the human and the robot are working together to assemble a vehicle’s seats in a real industrial context. The participants are required to cooperate smoothly by conducting a series of tasks. The robot participant carries out the part hand-over tasks and the human worker performs the assembly job. 

**Figure 4:**
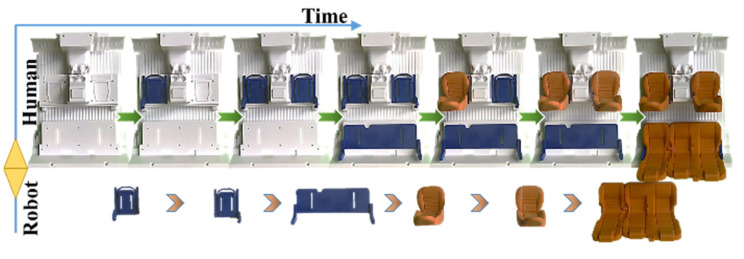
The vehicle seat assembly task in the experiment.

The task employed in this experiment derives from real industry assembly cases. The assembly task contains several sub-tasks; the detailed instructions are shown in Figure 4:

Sub-task 1: install the left seat holder;
Sub-task 2: install the right seat holder;
Sub-task 3: install the back seat holder;
Sub-task 4: install the left seat;
Sub-task 5: install the right seat;
Sub-task 6: install the back seat.

Sub-tasks 1–3 are independent of each other, which means Sub-tasks 1–3 can be carried out regardless of order. Sub-tasks 4–6 are independent of each other likewise. However, Sub-tasks 1–3 and Sub-tasks 4–6 are dependent so Sub-tasks 1–3 need to be finished before Sub-tasks 4–6 are carried out. After the completion of Sub-tasks 1–3, Sub-tasks 4–6 can be accomplished based on the human worker’s preference. 

In this project, the human and the robot cooperate by installing and delivering particular parts to accomplish the seat assembly. The human worker conducted and repeated the assembly procedures to demonstrate assembly preferences. The robot observed and learned the ongoing action intentions at the beginning phase. Subsequently, the robot constructed the collaboration knowledge and inferred the predicted actions. Afterward, the human worker sought the next part needing to be installed from the robot implicitly. The human worker reached out for the part when it was delivered by the robot, and proceeded with the following task. All the parts requirements and the delivery timing were conveyed indirectly, and the robot fulfilled its job without explicit commands. The assembly variances and noises were diminished by the prediction system. In other words, the robot needed to figure out the human worker’s collaboration hints and correctly respond in line with the human worker’s future intentions. The framework of this project is expandable and not bounded by the types of these tasks. 

In general, the task allocation in the collaboration depends on many factors, such as the attributes of the parts and the capabilities of the participants. In this case, the assembly process is adapted to establish robot-assisted collaboration. That is, the human worker handles the assembly maneuver in a guiding role and the robot compliantly handles the delivery of the parts. The installation of seat holders and seats is fulfilled by human workers, while the corresponding components are reached for and delivered by the robot. For instance, a possible collaboration sequence could be listed as:

Step 1: The human worker starts to install the left seat holder on the chassis;
Step 2: The robot picks up the right seat holder and delivers it to the worker;
Step 3: The human worker finishes the installation of the right seat holder;
Step 4: The robot picks up the back seat holder and hands it to the collaborator;
Step 5: The human worker concludes the installation of the back seat holder;
Step 6: The robot grabs the left seat and passes it to the human worker;
Step 7: The human worker finishes the installation of the left seat;
Step 8: The robot picks up the right seat and hands it to the human worker;
Step 9: The human worker completes the installation of the right seat;
Step 10: The robot grasps up the back seat and provides it to the human worker;
Step 11: The human worker conducts the installation of the back seat.

It should be noted that this experiment setting is designed for robot-assisted collaborative assembly. In this assembly assignment, the human worker governs the tasks in general while the robot mainly follows the human’s lead. This specific setting is established based on the current SME assembly scenario and customized human comfort considerations. Within this setting, the robot makes the subtle action prediction, provides essential assistance, and improves assembly efficiency. Our experiment setting takes the part prediction and delivery as the key collaborative action. The human worker needs 6 parts to accomplish the seat assembly task, which means 6 collaborative action predictions in one assembly instance. Each assembly instance takes 3–4 min to complete, with some individual variations. In addition, the human worker can focus on the assembly task and save the cost of robot action prediction. This setting also benefits the collaboration safety in some way.

### 3.4. The Control System Design and Safety Strategy

**Figure 5:**
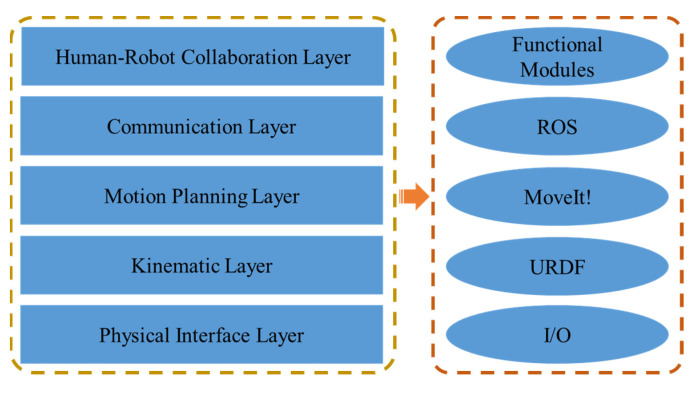
The structure of the control system.

The structure of the control system is shown in Figure 5, the corresponding software is described on the right side of the figure. As shown in Figure 5, the control system consists of five layers, which establish vital flexibility and extendibility for complex projects. The physical interface layer manages the connections with the hardware through various I/O interfaces. The kinematic layer handles the robot model through the URDF [49] file and builds the kinematics for future motion planning. The motion planning layer generates the proper trajectories by the MoveIt! [50] package and executes the joint trajectories to low-level hardware controllers. The communication layer is the basis of the system. Many libraries are embedded in the ROS package to speed up the developing course. It offers the information interchanging ability for customized modules in different layers via a predefined protocol. In the human-robot collaboration layer, many critical modules and built-in functions can be deployed for any specific task. In general, the control system can provide a modular platform for building modern robotics applications. 

We designed the robot control system based on the MoveIt! package which is integrated with the ROS. The control system allows the robot to construct the workspace environment setting, generate motion plans, and carry out the motion plans according to the change in the workspace. In the human–robot collaborative assembly assignments, the path planning performance directly affects the collaboration quality. The Open Motion Planning Library (OMPL) [51] in the ROS configuration is deployed to handle the trajectory planning jobs. There are many state-of-the-art planners in the OMPL collection, and the optimal path planner should be adopted to improve the robot’s efficiency and reliability for different collaborative scenarios. 

We conducted several delivery tests to compare the performance of different planners on the robot. Given the same task in the sharing workspace, the robot runs different planners multiple times to verify the planner’s performance. Finally, considering the planning path length, computation time, and execution time, we choose the fast marching tree (FMT) [52] planner for the robot to pick up parts and deliver them to its human coworker based on the result of the tests. 

During the assembly procedure, the robot drives its arm from the initial position to the inventory position, then picks up the desired part and delivers it to the human at the appropriate position, and eventually returns to the initial position. The grasp orientation in the part picking up position is set to downward, while the initial position remains the same during the assembly process. The robot’s motion paths are generated by the planner FMT. 

Safety for humans in our collaborative assembly procedure is ensured by several strategies and methods. First, we impose speed limits on the robot and ensure that it stays out of the human safety zone. When moving close to the human, the robot is allowed to move partially at a speed that is slow enough to raise no risk to the human worker. This collision avoidance strategy fits our project well enough and provides greater flexibility than the global constrained safety methods. In addition, collision avoidance is not always possible in the assembly scenario. Safety is a built-in feature of the robot we use. The soft padded arms and floating-plastic-covered skeleton combined with force-sensing control ensure the safety of human coworkers in the shared workspace. The material wrapped in the robot absorbs the unexpected impact, and the collision sensing system maintains the power and force below injury thresholds. Once the collisions were detected, the control commands to stop the robot were executed immediately. Furthermore, to deal with unpredictable events, emergency stops are equipped within easy reach in the shared workspace. If the emergency stop is triggered, the entire system will be overridden and shut down. The safety of human-robot collaboration is the top priority and all the strategies are just a small part of the safety-related system developed. 

## 4. Experimental Results and Discussion

In this part, we describe several experiments to evaluate results for the proposed model in the learning from demonstration, the predicted actions generation, and the following human-robot collaboration in the unlabeled assembly manufacture process. Then, we compare the performance of the proposed model with the speech commanded method to demonstrate the efficiency and smoothness of our strategy in the industrial human-robot collaborative assembly scenario.

### 4.1. The Result of Learning from Demonstrations and Action Prediction

The model uses visual data as the input features to learn spatial and temporal information. The model was trained with our human demonstration dataset. During the demonstrations, human workers perform the parts assembly process for the robot without explicit definition. The dataset is separated into the training part and the testing part. The testing dataset was used to test the accuracy and generalization of the proposed model. The performance was evaluated by predicting actions of different lengths from input video clips. With the proposed model, the robot can monitor the human worker’s actions, learn the human worker’s intentions from the movements, and understand the ongoing assembly tasks.

**Figure 6:**
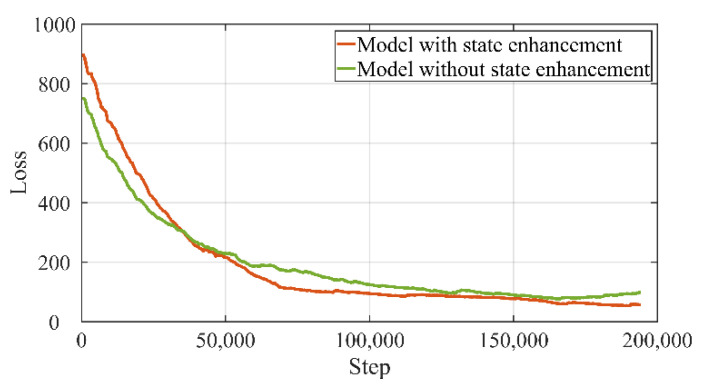
The training process and loss comparison of the learning from demonstration.

In the model training experiments, the training results of different configurations are compared. The loss of the training process is shown in Figure 6. The solid orange line represents the proposed model’s performance and the green line denotes the performances of the model without state enhancement. As shown in Figure 6, the proposed model outperforms the corresponding non-enhanced model during the experiments. It can be seen that the loss has decreased further than the model without state enhancements. At the same time, the converge time of the proposed model increases since more network parameters need to be tuned. Furthermore, the proposed model can predict a longer time span than the previous model, which is better for the following collaboration process. Evaluations of the testing dataset show that the proposed model mimics human prediction performance, and indicates the capacity of the network to predict the actions and timing of the near future assembly tasks. Since the model with state enhancement can learn and abstract more spatiotemporal information from human demonstrations, it will be applied in the following sections to generate predicted actions.

**Figure 7:**
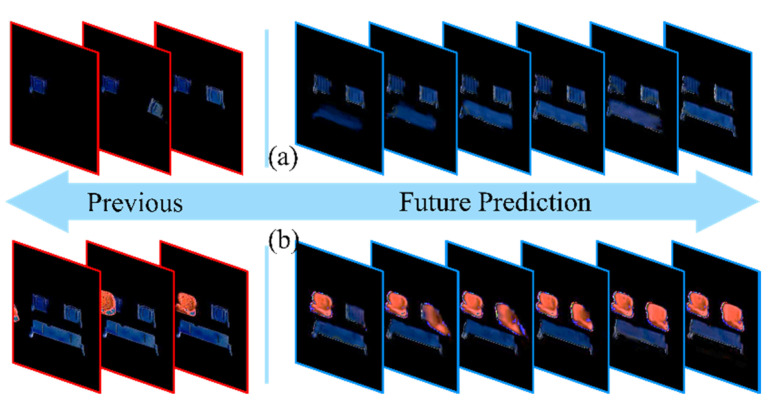
Prediction of the back seat holder installation (**a**), and the right seat installation (**b**).

In order to validate the learning and prediction performance of the proposed model, we applied another unknown demonstration to examine and generate the predictions. Figure 7 shows some prediction results in the assembly process. The red square denotes the provided previous input sequence, and the blue square is the future predicted sequence according to input.

It can be seen that the first predicted actions are the back seat holder being installed after the right seat holder assembly in the long-term prediction, as shown in Figure 7a. The action sequence is developed smoothly with vital spatial information. Normally, prolonged predictions are hard to generate due to the loss of spatiotemporal information. The prediction output here is fairly clear in the long-term regeneration, which reveals the advantage of state enhancement of the proposed model.

Likewise, the second actions are the right seat being assembled following the left seat installation. As can be seen from Figure 7b, the model processes the gist information from previous actions and renders the suitable video reconstruction. Compared to the human demonstration, the results indicate that the proposed model learns the essential information from the human actions and predicts the future actions with correct timing. To be more precise, the model can learn essential spatiotemporal information from previous human actions and make reliable predictions from both operation and timing perspectives.

The clear prediction results prove that the model can make accurate predictions during the assembly process without predefined tasks.

### 4.2. The Tracking Result of the Predicted Part

After obtaining the prediction output based on the neural network model, the potential human intention is also embedded in the predicted action sequences. The human intention has to be converted into the equivalent robot control command for the collaboration. Hence, for the future human–robot collaborative assembly, the specific part needs to be tracked and recognized at the correct time step.

**Figure 8:**
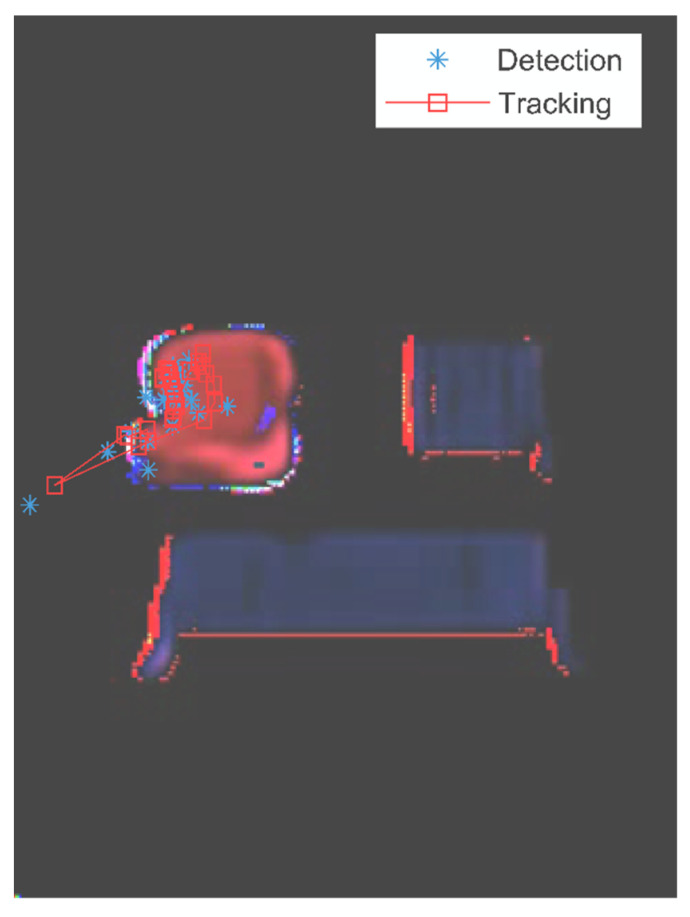
The tracking result of the intended left seat part.

Since the human intentions are nested inside the assembly motion of parts, we applied the EKF to track the intended part in the prediction motion sequence. For instance, the left seat is intended to be installed following the installation of the back seat holder. Figure 8 shows the assembly motions of the predicted left seat part in overlaying layers. The measurements of the left seat are marked by blue asterisks, and its motion trajectory is noted by a red line with the square marker. The results show that the estimations are matched with the assembly process, and the EKF-based method detects and tracks the potential left seat part successfully.

**Figure 9:**
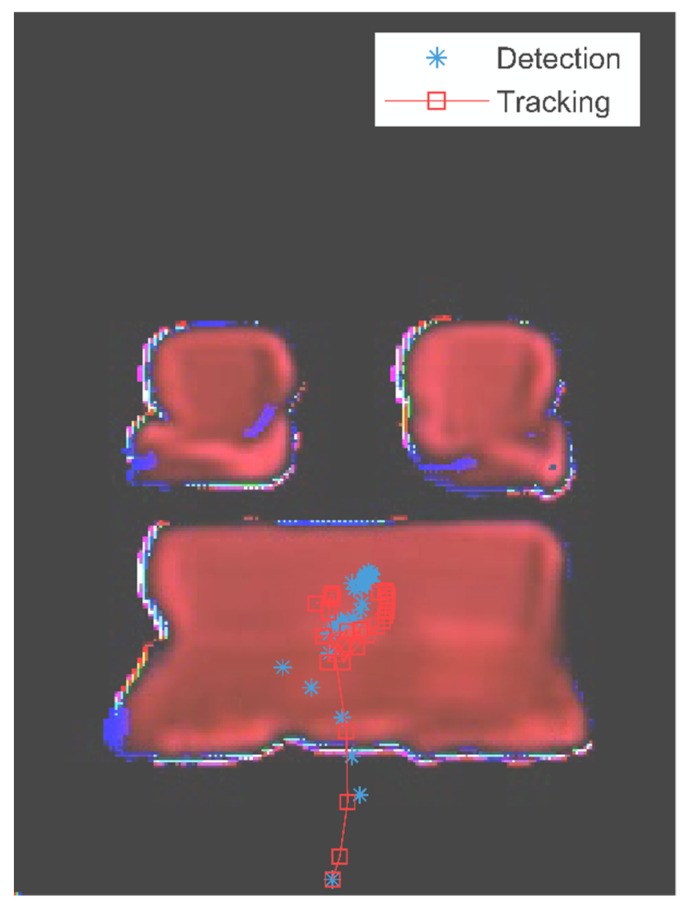
The tracking result of the back seat part.

The EKF method can make stable estimations of the tracked parts by weighting the measurement and estimation. It uses the measurements to readdress the estimation states for the filtered tracking, and output the estimation based on early states if no measurements occurred. Figure 9 shows the result of tracking the back seat in the predicted assembly process. The tracking trajectory is marked by the red line with the square sign as well. The trajectory has several twists due to the measurement noise during the assembly process. 

The tracking in our experiment is based on the part motion within the shared workspace, thus the misplacement of the parts is under control and the tracking errors are relatively small. In addition, more complex motion models and attributions of other parts can be used to reduce the tracking errors if needed.

### 4.3. The Tracked Part Dataset Building Process

Since we already concluded the future action sequence and captured the intended assembly part, obviously this precise part should be recognized in the next step and fed into the robot control system for preparation of assembly assisting executions. Before that, we need to build a large dataset for the following neural network’s training procedures. 

When we want to apply machine learning models in real industry scenarios, the desired data resource plays a crucial role in these data-driven [53] approaches. Data are at the core of the machine learning tasks. The machine learning model extracts latent features, establishes the implicit map between the input and the target, and validates its performance from the given training dataset. However, building the required dataset is a non-trivial mission in the machine learning procedure. In real industry projects, the dataset collection process is likely to become complicated and the data annotation practice tends to be time-consuming. In addition, constructing a high-quality dataset generally requires expert knowledge to collect, assess, and organize the chaotic data for the demanding CNN model. 

**Figure 10:**
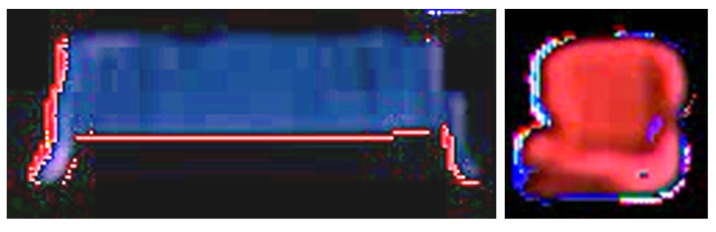
Typical data samples were collected from prediction videos.

**Figure 11:**
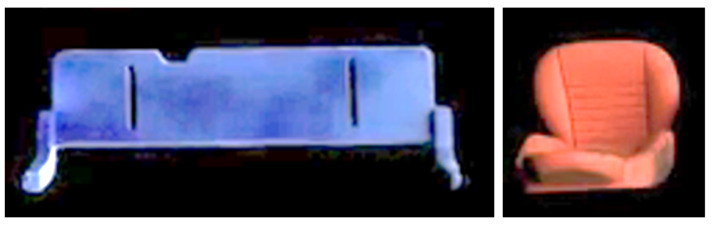
Typical data samples were sampled from demonstration videos.

For this specific assembly scenario, the potential data-driven model shares the same challenges. First and foremost, enough data need to be collected effectively. Here, we take advantage of the previous tracking model to alleviate the pain of data collection. During the tracking process, we tracked a lot of images for desired parts in the prediction video sequences, these images contain the independent parts in an organized way. In addition, the travel behavior of these parts is slightly different, showing different angles and positions in the tracked images. These various images can be cropped out during the intermediate tracking process, as shown in Figure 10. Meanwhile, the collection of various images during tracking can serve as a data augmentation [54] approach. Furthermore, there is another path to enrich this data collecting process. Since the output of the prediction model shares the same format as the demonstration input, the tracking model can be applied directly to the demonstration video sequences. Likewise, this procedure also provides a lot of independent part images for the dataset construction. As shown in Figure 11, the samples from the demonstration video are more sharp and clear than the samples from prediction sequences. These samples provide more clean data and more variations for the dataset, which is necessary to achieve the robustness of the desired model by increasing the generalization ability.

After gathering the raw data from the shared workspace, the samples already covered all use cases and vital information. Generally, there is plenty of work that needs to be done in the data labeling [55] process. However, the image data of this assembly process can be labeled semi-automatically. Since the raw data were captured during the tracking process, the obtained parts are organized in a continuous procedure. All the data samples can be labeled based on assembly sequence and can be processed in a batch-by-batch way. 

During the prediction tracking process, the images of each tracked part are mapped to a specific label that corresponds to the correct part name for the following assembly action. As in the prediction tracking process, the images of each tracked part in the demonstration tracking process are assigned to the corresponding part name as well. Here, we treat the demonstration input as the prediction output cautiously. Each prediction yields a series of tracked images, and these images may not follow the normal assembly sequence, but each part and its labels do not change at each step of the assembly process. 

Therefore, we only need to focus on the beginning and end of each image series. If the beginning and ending parts are the same, then the whole image series can be labeled without hesitation. If the beginning and ending parts are different, then it indicates that a new part was assembled after the previous part was assembled during the assembly process, and both parts should be labeled individually. 

Table 2 shows the samples we obtained with tracking predicted and demonstration clips based on semi-automatic labeling. These samples are obtained from several demonstrations and predictions. The constructed dataset is representative of all the intended parts. 

### 4.4. The Recognition Result of the Intended Part

After the tracking and label process is finished, the dataset can be used to train a CNN to recognize the predicted part. The label represents the human intention in the next assembly step. When the human operator is assembling the product, the predicted images are sampled and all the images will be converted to labels, and these labels will be transferred to the robot to carry out a real collaboration task in the subsequent process.

The robot needs to learn through enough data to obtain the correct part based on human intentions. The model structure and corresponding parameters are displayed in Table 3. The images from the dataset were resized to 128 × 128 and fed into the network. There are five convolution layers and two fully connected layers before the classifier.

In the experiment, the model was trained by different dataset configurations, one with the demonstration dataset and one with the combined dataset. We use 70% data to train, 20% data for validation, and 10% data to tune the CNN model in the same way. The samples shown in Table 2 include samples from demonstrations and predictions, and the model trained from different datasets will be applied to the real subsequent assembly predictions to recognize human intention parts. As the result of our experiment, the model with a combined dataset achieves higher validation accuracy in a relatively larger training iteration. Here, we use the combined dataset configuration trained CNN model for better performance.

**Figure 12:**
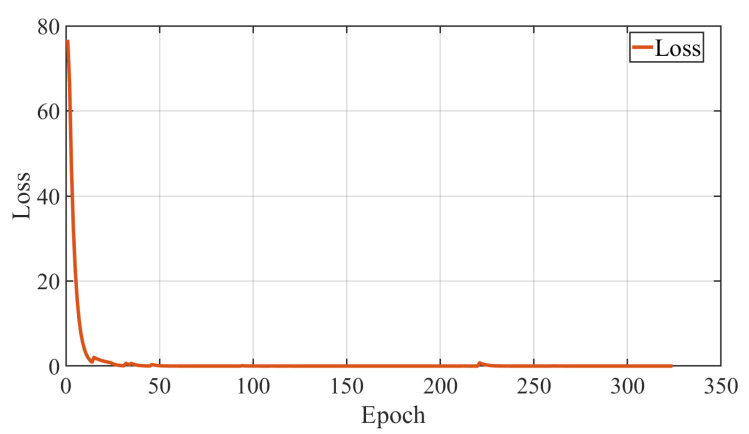
The loss function change of the proposed recognize model.

**Figure 13:**
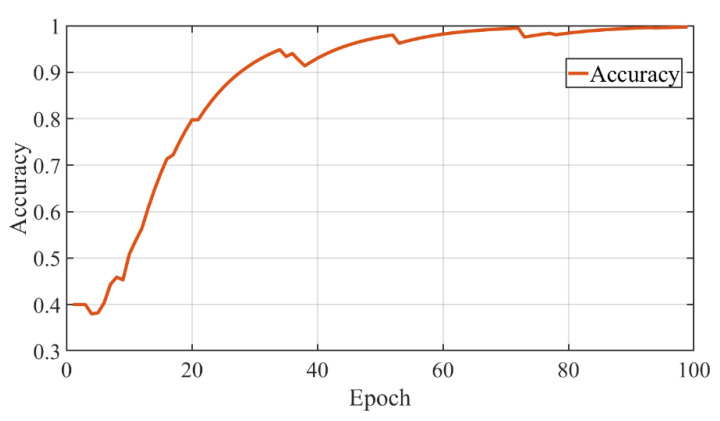
The accuracy of the intended part recognition model.

We compare the performance of the model trained only from the demonstrations dataset with the model from the combined dataset. The model was trained for 350 epochs, and the validation was carried out every 1000 iterations. With each saved best model, the performance test procedure can be fulfilled. Figure 12 shows the loss function change of the combined dataset training process. From Figure 12, we can see that the model converges at around 65 epochs. In terms of learning accuracy, the model based on the combined dataset achieves a higher score in the experiment, as shown in Figure 13.

The recognition accuracy of the selected model is higher than 99% during the following test scenarios. This model can handle human intention parts recognition successfully for the prediction actions in our assembly collaboration task. The specific intention part will be fed into the robot control system during the following collaboration process.

### 4.5. The Result of Human-Robot Collaboration

After the completion of learning from the human demonstration process, predicting human future action, and obtaining the correct required part, the human-robot collaborative assembly routine can be established. 

**Figure 14:**
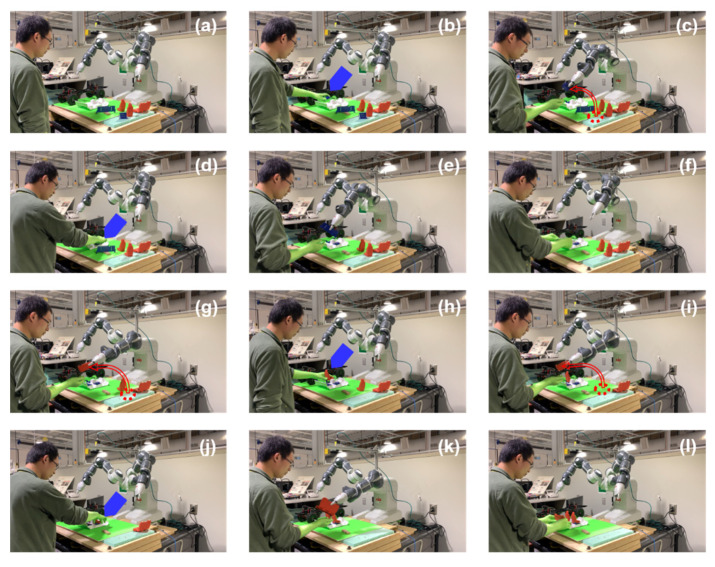
Human-robot collaborative assembly process in real experiment scenario. (**a**) Initial position; (**b**) human worker installs the left seat holder; (**c**) robot delivers the right seat holder to human; (**d**) human worker installs the right seat holder; (**e**) robot delivers the back seat holder to human; (**f**) human worker installs the back seat holder; (**g**) robot delivers the left seat to human; (**h**) human worker installs the left seat; (**i**) robot delivers the right seat to human; (**j**) human installs the right seat; (**k**) robot delivers the back seat to human; (**l**) human installs the back seat.

While launching the human-robot collaborative assembly procedure, the proposed model is deployed upon ROS to assist the human worker to install vehicle seats. After learning the customized assembly information from the human demonstration, the robot infers the human worker’s future intention and provides the corresponding required part. The intended part’s information, such as location and destination, is transferred to the robot’s controller. Based on that, the robot finds the part in the inventory, plans the proper motion trajectory, grasps the correct part, and delivers it to the shared workspace. The collaborative vehicle seat assembly procedure is shown in Figure 14.

From Figure 14a,b, it can be seen that the human worker installs the left seat holder into the vehicle floor part without explicit instructions. Based on the previous human actions and current assembly status, the early prediction action is planned according to the proposed model, and the robot grasps the right seat holder and delivers it to the human worker, as displayed in Figure 14c. In the same manner, the human worker installs the right seat holder in the following step as shown in Figure 14d, then the robot proactively delivers the back seat holder part to its cooperator, as presented in Figure 14e. Subsequently, the robot assists its partner to accomplish the vehicle seat assembly according to the human worker’s preference in a smooth way. Note that the blue and red arrows highlight the assembly details in the corresponding step.

**Figure 15:**
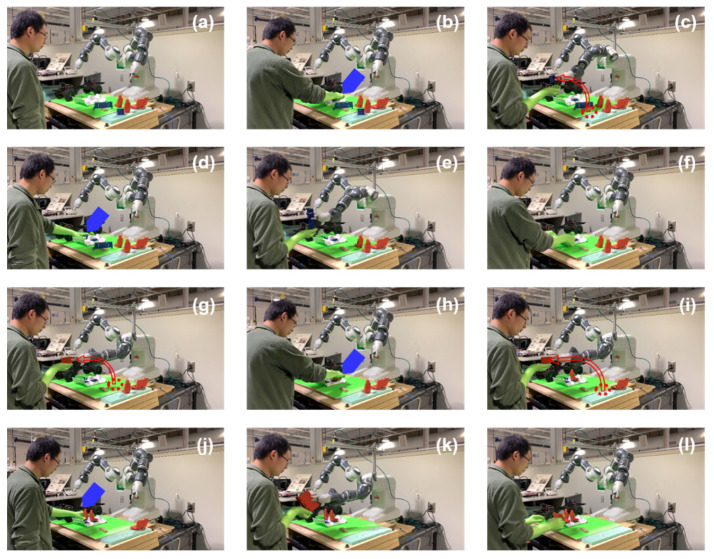
Human-robot collaborative process for a different assembly preference. (**a**) Initial position; (**b**) human worker installs the right seat holder; (**c**) robot delivers the left seat holder to human; (**d**) human worker installs the left seat holder; (**e**) robot delivers the back seat holder to human; (**f**) human worker installs the back seat holder; (**g**) robot delivers right seat to human; (**h**) human worker installs the right seat; (**i**) robot delivers the left seat to human; (**j**) human installs the left seat; (**k**) robot delivers the back seat to human; (**l**) human installs the back seat.

In a real assembly scenario, the same product can be assembled in a different style according to the human worker’s preference. We also test the model on a different assembly option in our experiment. The result is shown in Figure 15 in the same manner as Figure 14. As shown in Figure 15a,b, the human worker installs the right seat holder first under the monitoring of the robot. In Figure 15c, the robot analyzes the human actions, plans the following tasks based on the prediction model, and executes the left seat holder delivering action. The assembly details are marked by the blue and red arrows. Then, the human worker finishes the left seat holder installation in Figure 15d, and the robot plans and executes the back seat holder delivery process in Figure 15e. Since there are different seat holder installation arrangements, the seats need to be set up accordingly. After the human worker installs the back seat holder shown in Figure 15f, the robot grasps the right seat and delivers it correctly based on the previous assembly process as shown in Figure 15g. From Figure 15h–l, the robot assists the human worker in other seat installing procedures actively in a matching manner. 

As demonstrated in Figure 14 and Figure 15, the robot delivers the correct part at the right time during the collaboration process, and the human-robot collaborative assembly process was achieved successfully. The results show that the robot can proactively assist the human worker in the assembly manufacturing procedure. Furthermore, the deployed model can adapt to the partner’s customized assembly style and collaborate on various assembly forms fluently.

### 4.6. Comparison with Speech Commanded Assist Robot

To verify the performance of the early prediction-based approach, we have implemented a more straightforward method in the same assembly scenario. In a human-robot collaborative assembly context, the human worker can express his intention directly through natural language. The robot can interpret the human worker’s intentions through language and then deliver the corresponding part to the human. For this setting, the natural language commands are the bridge between robot and human worker. This is similar to the human workers’ cooperation procedure. A human gives the speech instruction to the collaboration partner, the partner understands the instruction and arranges the corresponding action, then the partner executes the action. In this situation, the human worker serves as the leader and delegates the desired task to his partner, the assisting robot. 

We adopted the Google Cloud speech recognition platform [56] to process the natural language commands. All the speech instructions are sent to the cloud and converted from speech to command text. The human assembly intentions are represented by the commands extracted from the natural language. Once the speech is decoded, the robot assists the human worker by performing the correct tasks based on the customized assembly sequence. For example, when the human worker required a specific part from the robot, he gave the part’s name, ‘left seat’, to the speech interface, and the speech recognition platform processed the voice and outputted the parallel text commands. Then, the recognized commands were fed into the robot controller, the robot picked the desired part, planned the trajectory, and delivered it to the human for assembly. During the collaboration, human workers can change task sequences according to their preferences and define customized assembly procedures. These various actions are similar to the prediction-based approach.

However, after receiving the human’s instruction and planning the potential action, the robot locates and delivers the required parts within a relatively longer time span than the prediction-based method. The actual intention will be fulfilled after the whole commands are interpreted by the robot and the trajectory optimized by the robot planner. Furthermore, the human worker needs to pay extra attention to things other than his own tasks. This additional work introduces a tedious burden in the collaboration, which is against the safety and comfort expectations in the long run. 

We invited another six individuals (one female and five males) within an age range of 21–35 as participants in the experiments. The participants included students and researchers with non-directly related backgrounds. Each of them collaborated with the robot to conduct the assembly tasks twice using the proposed prediction-based HRC approach and speech commanded approach. Data from these collaborations were collected and exploited for further model performance evaluation. 

**Figure 16:**
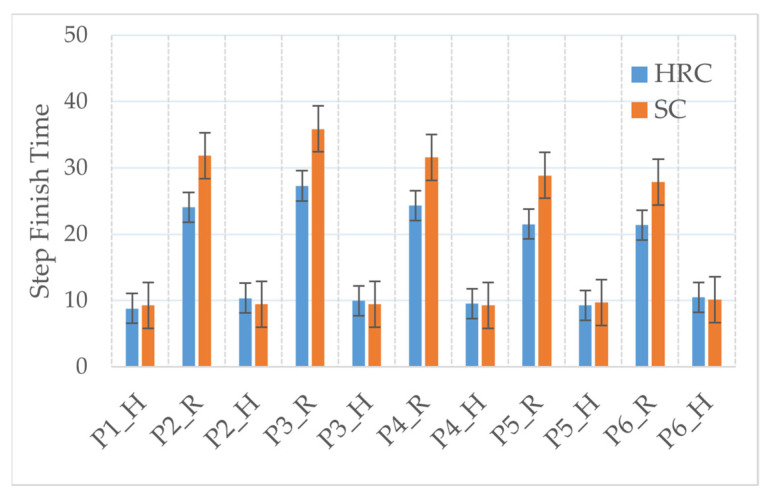
Step finish time comparison between human–robot collaboration with early prediction and speech commanded assistance.

Figure 16 shows each step finish time for two methods in the vehicle seat assembly experiment. P1–6 stand for the assembly part name, while H and R denote the task handled by human worker and robot, respectively. As shown in Figure 16, when the assembly subtask was managed by the human worker, the finish times for every sub-task are similar to each other for both collaboration methods. However, there is a noticeable difference in the step finish time between the two approaches. It can be seen that the HRC with the early prediction method takes less time than the speech commanded method. The prediction-based approach performs a faster collaboration for the experiment scenario. The speech commanded assistance robot needs more time to receive the speech and decode the commands from the cloud platform, which introduces a bigger idle gap during the assembly process and consequently decreases the smoothness of collaboration.

**Figure 17:**
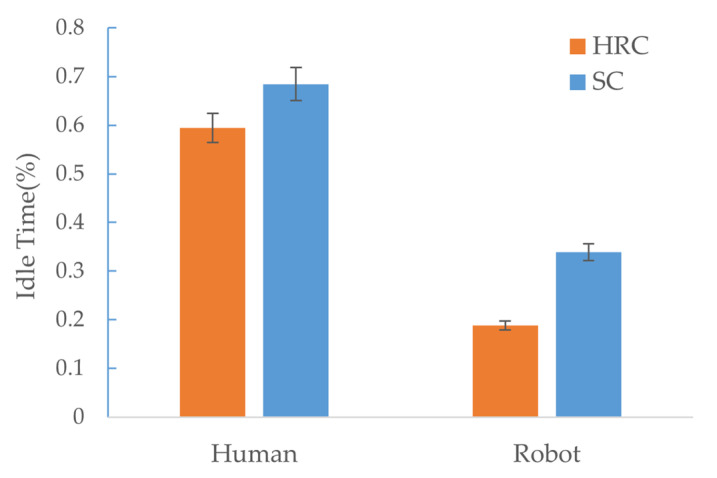
Human idle time and robot idle time comparison between human-robot collaboration with early prediction and speech commanded assistance.

To evaluate the fluency of the human–robot collaboration, we compare the human idle time and robot idle time [57] between the prediction-based HRC approach and the speech commanded approach, as shown in Figure 17. The human idle time and robot idle time are applied to obtain the objective measures. Human idle time refers to the percentage of human inactivity in the total task time. Human idle time occurs in situations such as the human waiting for the robot to finish its action. Similarly, the robot idle time refers to the rate of robot inactivity in the total task time. Robot idle time happens due to waiting for input, the computing of motion plans, waiting for prior human action, etc. All these measures are calculated based on the intervals of assembly action. Figure 17 shows that the human idle time is relatively high in the speech commanded approach because of the extra time required for speech command recognition and robot motion planning. Meanwhile, robot idle time is also relatively high, due to a similar reason. On the contrary, the idle time of both the human and the robot is comparatively low in the prediction-based HRC approach, since there are more concurrent activities and less time taken for the human to wait for the robot to deliver the desired part to continue the assembly assignment. 

Collectively, it appears that the robot idle time is much lower than the human idle time, suggesting effective use of the robots. The higher human idle time indicates that relevant issues such as human safety and comfort are considered in task allocation and can be reduced in future studies. Moreover, compared with the speech commanded approach, the HRC approach reduced human and robot idle time values by 34.5% and 58%, respectively. This indicates that, although the human idle time is reserved for safety reasons, the proposed prediction-based approach achieves fluent collaboration and efficient resource utilization in the collaborative assembly scenario.

**Figure 18:**
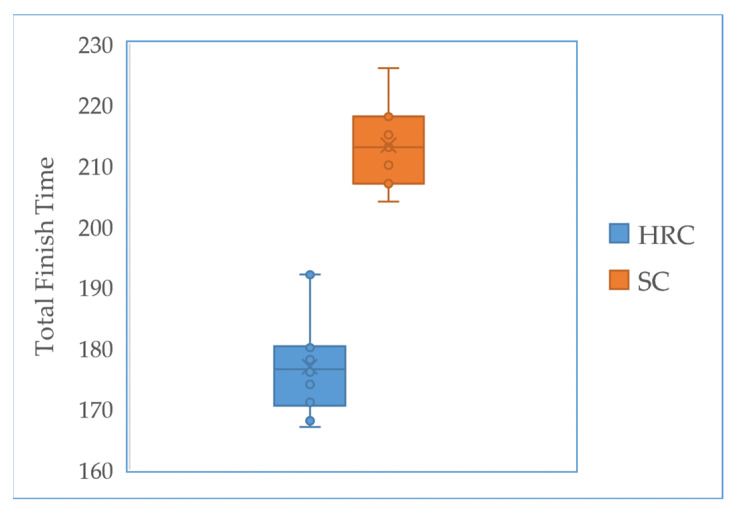
Total finish time comparison between human-robot collaboration with early prediction and speech commanded assistance.

Here, we compare the total task finish time between the prediction-based HRC approach and speech commanded approach, as shown In Figure 18. The task time in the proposed method is shorter than the speech commanded method. The idle time in the speech commanded system is significantly longer than the proposed model. The early prediction-based HRC method takes an average of 36.43 s less than the speech commanded assistance method. This result indicates that the early prediction-based model has better performance and more smooth cooperation, which benefits the human-robot collaborative scenario.

## 5. Conclusions

Early prediction plays an important role in human-robot collaboration. This paper presents a prediction-based framework to understand, learn, and assist human coworkers in the HRC assembly scenario. The proposed model was implemented on a robot to help in the manufacturing assembly process by delivering parts for installation. Experimental results show that the model is effective in learning from demonstration, and the robot can predict the human partner’s future intentions and then naturally deliver corresponding parts to assist humans proactively in real time. The early prediction-based intention aware robot has better cooperation fluency, shorter suspended time, and higher collaboration comfort, compared to the traditional passive commanded one. These outcomes verify the effectiveness of the human intention prediction-based framework and demonstrate the importance of future task awareness in human-robot collaboration. In addition, the predictions inferred from human demonstration can be customized to different human partners according to their unique working styles. Compared with existing methods, the developed approach can help alleviate the need for heavy handcrafted models and complex settings. This model also proposes a potential way by which human coworkers can personalize the robot and let the robot assist them in the way they prefer. The proposed framework can serve as a core module in future human-robot collaboration applications and extend the exercise of more intelligent robot systems. There are limitations in the proposed method for predicting human intentions in some occlusion scenarios. The prediction results are affected by the limited workspace due to the view field of the camera. For instance, human workers sometimes need to clean the parts in areas that may not be covered by the camera during assembly manufacturing. Further developments of this work include more complex practices in industrial applications, a wearable-sensor-equipped system that is less susceptible to occlusions, and more methods to ensure safety and improve efficiency simultaneously.

## Figures and Tables

**Table 1:** The comparison of different methods in human action prediction.

Method	Advantages	Disadvantages
Fuzzy method [33]	Simple model, small dataset requirement	Weak generalization ability, predefined rules.
Hidden Markov model [26]	Model clarity, training flexibility, small dataset requirement	Hard to tune the parameters, weak dynamics analysis, short-term prediction
Hidden semi-Markov model [28]	Model clarity, training flexibility, small dataset requirement	Hard to tune the parameters, short-term prediction
Extreme learning machine algorithm [35]	Model clarity, small dataset requirement, high accuracy	“Black box” nature, short-term prediction
Convolutional neural network + LSTM [32]	High flexibility, effective in heavy data flow	“Black box” nature, classification based, low accuracy
State-enhanced ConvLSTM	End-to-end, undefined task learning, training flexibility, long-term prediction, customized ability	“Black box” nature, high resource requirements

**Table 2:** The dataset of the tracking process.

Parts	Demo	Prediction	Sum
Left Seat Holder	2500	1000	3500
Right Seat Holder	2500	1000	3500
Back Seat Holder	2700	1100	3800
Left Seat	2500	1000	3500
Right Seat	2500	1000	3500
Back Seat	2700	1100	3800

**Table 3:** The structure of the CNN model.

Layer	Stride	Input size	Filter size
Conv	1	128 × 128 × 3	5 × 5 × 32
Max Pooling	2	128 × 128 × 32	2 × 2
Conv	1	64 × 64 × 32	3 × 3 × 32
Max Pooling	2	64 × 64 × 32	2 × 2
Conv	1	32 × 32 × 32	3 × 3 × 64
Max Pooling	2	32 × 32 × 64	2 × 2
Conv	1	16 × 16 × 64	3 × 3 × 64
Max Pooling	2	16 × 16 × 64	2 × 2
Conv	1	8 × 8 × 64	3 × 3 × 128
Max Pooling	2	8 × 8 × 128	2 × 2
FC	1	1 × 1 × 2048	2048 × 512
FC	1	1 × 1 × 512	512 × 256
Softmax	1	1 × 1 × 256	Classifier

## Data Availability

The data presented in this study are not publicly available at this time but can be obtained from the authors.
